# Preventing kidney injury using carbon dioxide (KID trial): trial protocol for a multicentre randomised controlled trial

**DOI:** 10.1136/bmjopen-2025-111752

**Published:** 2025-11-28

**Authors:** Athanasios Saratzis, Neha Rasheed, Dania Aguirre, Patrick Coughlin, Athanasios Diamantopoulos, Lindsay Bearne, Nicholas M Selby, Cassandra Brookes, Shaun Barber, Carla Richardson, Hannah Gilbert, Roland Schueller, Despina Apergi, Keith Jonathan Harris

**Affiliations:** 1 Cardiovascular Sciences, University of Leicester Medical School, Leicester, Leicestershire, UK; 2 University of Leicester, Leicester, Leicestershire, UK; 3 Vascular Surgery, Leeds Vascular Institute General Infirmary, Leeds, UK; 4 Guy’s and St Thomas’ Hospitals NHS Foundation Trust, London, UK; 5 Department of Population Health Sciences, King’s College London, London, UK; 6 St George’s University of London, London, UK; 7 Centre for Kidney Research and Innovation, University of Nottingham Faculty of Medicine and Health Sciences, Derby, Nottingham, UK; 8 Royal Derby Hospital, Derby, UK; 9 Leicester Clinical Trials Unit, University of Leicester, Leicester, UK; 10 Leicester Vascular Institute, University Hospitals of Leicester NHS Trust, Leicester, UK

**Keywords:** Vascular surgery, NEPHROLOGY, Clinical Trial, Acute renal failure

## Abstract

**Introduction:**

Peripheral arterial disease (PAD) commonly coexists with chronic kidney disease (CKD). Patients with symptomatic PAD often require endovascular revascularisation to relieve pain or salvage limbs. However, the iodinated intra-arterial contrast routinely used in these procedures is nephrotoxic, placing patients with CKD at increased risk of acute kidney injury (AKI) and long-term renal decline. Carbon dioxide (CO_₂_) delivered via automated injection is a potential alternative imaging contrast medium. This trial will evaluate whether using CO_₂_ instead of iodinated contrast reduces the risk of AKI and short-term renal function decline in this high-risk group.

**Methods and analysis:**

This is a multicentre, open-label, prospective randomised controlled trial across six secondary-care National Health Service (NHS) vascular surgery centres. A total of 174 patients with PAD and CKD undergoing endovascular intervention will be randomised 1:1 to receive iodinated contrast (standard of care) or CO_₂_ via automated injector (Angiodroid). All perioperative care will follow local NHS protocols.

The primary outcome is log serum creatinine at 2, 30 and 90 days postprocedure. Key secondary outcomes include: incidence and severity of AKI within 48 hours postprocedure, major adverse kidney events (death, dialysis or >25% estimated glomerular filtration rate decline) by 90 days, inpatient length of stay, procedural pain, quality of life, procedural success, reinterventions, acceptability and feasibility (patient/practitioner questionnaires) of using CO_2_, and cost-effectiveness (healthcare resource use analysis). A mixed-methods process evaluation will be undertaken with patients and clinicians.

**Ethics and dissemination:**

The trial has been approved by an NHS ethical review committee (24/WA/0332) and patients have been involved in trial design. Findings will be disseminated to participants, clinicians and the wider public through patient groups, lay summaries, social media, conferences, peer-reviewed journals and NHS policy channels.

**Trial registration number:**

ISRCTN23564393.

**STRENGTHS AND LIMITATIONS OF THIS STUDY**

- This is a multicentre, pragmatic randomised controlled trial conducted across six National Health Service (NHS) vascular centres, enhancing the generalisability of findings.
- The trial design incorporates both clinical and health economic analyses, allowing evaluation of effectiveness and cost-effectiveness within routine NHS care.
- Outcome assessments are based on objective, routinely collected biochemical measures, reducing measurement bias.
- A mixed-methods process evaluation enables detailed assessment of feasibility, acceptability and implementation factors for CO_₂_ angiography.
- The open-label design may introduce performance bias, although objective renal outcomes minimise its potential impact.

## Introduction

### Background and rationale

 When revascularising the lower limbs of people with peripheral arterial disease (PAD), iodine-based contrast medium is used to visualise arteries and allow operators to perform the procedure.^1 2^ This contrast medium, being nephrotoxic, can contribute to acute kidney injury (AKI), the most common complication after these procedures. One way to minimise AKI risk may be to use carbon dioxide (CO_2_) as a contrast medium, which is not nephrotoxic and does not contain iodine (CO_2_ is used widely in other invasive procedures, for example, laparoscopy).^3^ The value of using CO_2_ to reduce AKI in this context, however, has not been tested in a randomised setting.

AKI is the most common complication of endovascular (minimally invasive) revascularisation for PAD, especially in patients with pre-existing chronic kidney disease (CKD).^1^ Postintervention AKI in this context is strongly and independently associated with higher mortality, cardiovascular events and National Health Service (NHS) costs, in the short and long term.^4 5^ One of the key reasons why these patients develop AKI is the use of iodinated contrast medium, which is injected to visualise their arteries.^6^ CO_2_ can also be used as a contrast medium during such procedures; CO_2_ is safe to be injected intra-arterially and not nephrotoxic.^7^,^9^ The use of CO_2_, however, can be challenging due to the complexity of the procedure, imaging quality and pain during injection. Recently, automated intra-arterial CO_2_ injectors were developed. These new injectors overcome many constraints associated with injecting CO_2_ in arteries during revascularisation.^7^,^11^ This could lead to a shift in how arterial revascularisation is performed, preventing AKI and the major complications associated with it. Before this happens, the value of CO_2_ via automated injectors as a means of preventing AKI postendovascular revascularisation needs to be tested in a randomised trial. We also need to understand the acceptability, feasibility and factors that may influence the adoption/implementation of the technology in the NHS, as per National Institute for Health and Care Research (NIHR) and Medical Research Council guidance.^12^ This proposed randomised research will provide the necessary information to guide the implementation/adoption of this technology in the NHS, potentially leading to improved outcomes for patients and decreasing healthcare costs.

### Research questions

1. Does the use of CO_2_ via an automated injector during endovascular revascularisation reduce: (1) the likelihood of short- and medium-term renal decline? (2) The volume of iodinated contrast medium necessary to complete the procedure?
2. What is the acceptability, feasibility and the factors that may influence the adoption of this technology in the NHS from the perspective of patients and practitioners?
3. What are the costs of performing endovascular lower limb revascularisation using an automated CO_2_ injector, compared with the usual NHS standard of care (no use of CO_2_)?

### Objectives

#### Primary objective

Perform a multicentre prospective randomised controlled trial (RCT) to assess whether using an automated CO_2_ injector reduces the impact of endovascular revascularisation on renal function in patients with PAD and CKD by reducing the amount of nephrotoxic iodinated contrast medium necessary to complete the revascularisation.

#### Secondary objectives

Assess the acceptability, feasibility and the factors that may influence the adoption of this technology in the NHS from the perspective of patients and practitioners, record and report the overall costs of performing endovascular lower limb revascularisation using an automated CO_2_ injector versus the usual NHS standard of care.

## Methods and analysis

### Trial design

Multicentre, prospective, open-label, parallel-group RCT. The trial will also include a health economic evaluation to assess cost-effectiveness from both an NHS and a wider English societal perspective.

### Trial setting

Six NHS hospital sites in England providing arterial intervention services, which are already using the Angiodroid CO_₂_ injector. Preoperative and postoperative care will follow local protocols of care (pragmatic RCT).

### Inclusion criteria

- Age >18 years.
- Undergoing any peripheral arterial revascularisation with an endovascular component.
- Estimated glomerular filtration rate (eGFR) <60 mL/min/1.73 m^2^ at baseline or known established CKD (two separate eGFR readings <60 mL/min/1.73 m² in the last 3 months).
- Signed informed consent.

### Exclusion criteria

- Known allergy to iodinated contrast media.
- Individuals already on long-term dialysis at the time of screening.
- An inability to understand the languages in which the trial materials are provided.

#### Inclusion criteria for healthcare professionals involved in the parallel non-randomised implementation study

Clinicians who have performed the revascularisation procedure for the patient participants included in the RCT.

### Interventions

Participants will be randomised to either:

- *Control group*: standard iodinated contrast medium used during endovascular PAD revascularisation (standard of care).
- *Intervention group*: CO_₂_ contrast, delivered via a dedicated injector system (Angiodroid injector) as an alternative to iodine contrast.

All other procedural aspects remain consistent across groups and as per the usual standard of care (pragmatic RCT). Those in the intervention group will be allowed the administration of standard iodine-based intra-arterial contrast if deemed necessary by the operators in order to complete the procedure safely.

### Outcomes

Outcomes and assessments are based on national and international consultation with AKI experts, our national stakeholder/patient survey, patients’ opinions during our patient involvement work and our previous/ongoing randomised and observational research relating to renal decline in vascular patients.^1^

We plan to evaluate the impact on kidney function postendovascular revascularisation in the RCT arms both in the short term (AKI) and at 90 days (medium term). We will include creatinine measurements at 2, 30 and 90 days, powered to detect a clinically meaningful difference in serum creatinine (SCr) at 90 days. These three SCr measurements will also allow us to compare AKI incidence based on the Kidney Disease Improving Global Outcomes (KDIGO) definition of AKI between groups and calculate/compare the incidence of major adverse kidney events at 90 days (MAKE outcome). This will provide novel information on the clinical value of using the proposed intervention, addressing the relevant evidence gap and leading to adoption of the intervention in the NHS, for the immediate benefit of patients in this high-risk group. We have chosen not to use AKI incidence at 2 days as the primary outcome, as it does not allow us to evaluate medium-term renal injury and would also necessitate recruitment of a prohibitively large sample of patients, without adding clinical information that would aid translation to clinical care.

### Primary outcome

Log SCr measured from the blood sample taken at 2, 30 and 90 days (part of the standard of care) after endovascular revascularisation.

### Secondary outcomes

#### Key secondary outcome

Volume of iodinated contrast medium used during endovascular revascularisation.

The trial is adequately powered to assess both the primary outcome and the key secondary outcome. This will provide clinicians with high-quality definitive evidence and data necessary in order to adopt this intervention into their routine clinical practice.

#### Other secondary outcomes

1. Proportion of participants developing AKI (per stage of AKI) within 2 days (48 hours) of procedure based on the KDIGO definition of AKI-stages.
2. Proportion of participants developing MAKE by 90-day follow-up. A MAKE event is defined as either death, new requirement for dialysis or worsening kidney function, denoted by >25% decline in eGFR. Baseline eGFR will be defined as the most recent value before the procedure.
3. Time spent as an inpatient (days) since randomisation measured using patient medical records by a 90-day follow-up.
4. Pain experienced during the procedure was measured using a Visual Analogue Scale (VAS).
5. Quality of life (QoL) at 90-day follow-up was measured using the EQ-5D-5L (European Quality of life 5 Dimensions 5 Levels questionnaire) and Social Function - 46 items (SF-36) questionnaires.
6. The proportion of revascularisation completed as planned, measured using patient medical records at one time point.
7. Number of surgical or endovascular reinterventions measured using patient medical records by the 90-day follow-up.
8. Perceived acceptability, feasibility and factors that may influence the adoption of CO_2_ during each revascularisation by the practitioners/participants were measured using the Theoretical Framework of Acceptability (TFA) Questionnaire at the 90-day follow-up for patients, and postprocedure (administered within 3 days of the intervention) and cross-sectional questionnaires (administered after a few months into recruitment) for practitioners.
9. Costs associated with each procedure (health economic analysis) measured using a Healthcare Resource Use questionnaire over a minimum 90-day period (end of clinical follow-up).

Fluid therapy and medical care per patient will be documented for both groups before and after the procedures, using a standardised data collection system, based on our completed NIHR-Research for Patient Benefit funded Community and Hospital cAre Bundle to improve the medical treatment of severe cLaudIcation and critical limb iSchaemia (CHABLIS) study.^13 14^ Further, all equipment/devices used in each procedure will also be collected, and the duration of the procedure will be documented, to allow an in-depth health-economic analysis (additional package funded separately).

### Participant timeline and schedule of events

Participants will be screened and consented up to 15 days prior to the index procedure. Baseline assessments include medical history, demographics, height, weight, baseline SCr and patient-reported outcome measures relating to QoL (using the patient-preferred EQ-5D-5L and SF-36 forms).

Randomisation occurs after consent and baseline measurements, allocating participants 1:1 to iodinated contrast medium or CO_2_ via an automated injector. The index procedure (endovascular revascularisation) will be performed within 15 days of consent.

Follow-up assessments will coincide with routine NHS care at Days 2, 30 and 90 after the procedure. These include SCr measurements, repeat patient-reported outcome measures (Day 90), pain scores (VAS during the index procedure) and healthcare resource use questionnaires (Day 90).

An optional qualitative interview will be offered 3–4 weeks postprocedure to a purposive sample of participants and practitioners to explore intervention acceptability and implementation factors.

### Statistical considerations

#### Sample size calculation

174 patients (87 per group) provide 90% power to detect clinically meaningful differences. Based on our preliminary data (observational AKI study of 2021 patients with PAD/CKD treated in three sites and a pilot study on the use of CO_2_ in PAD procedures),^78 15^,^17^ we expect the SD of log SCr values at 2, 30 and 90 days to be 0.25 log (µmol/L). We assume the correlation of the baseline, 2, 30 and 90-day measures to be 0.67, which is conservative. With these assumptions, a sample size of 156 patients (78 per group) would have a 90% power to detect a mean difference of 0.075 log (µmol/L) in SCr values (effect size of 0.3) at 2, 30 and 90 days postsurgery, between intervention and control groups with an alpha value of 0.05. Allowing for a 10% drop-out, 174 will be recruited. This sample size will also allow us to assess for a 20% reduction in the volume of iodinated contrast used in those having the intervention versus control (median of 70 mL of iodinated contrast used in our published series without CO_2_). In order to ensure the population remained truly per intention-to-treat (ITT) for the primary outcome, multiple imputation will be implemented to deal with missing data in instances where participants are missing any of the SCr values. Values will be predicted using a multivariate normal regression equation with 20 imputations being used.

#### Recruitment

Potential participants will be identified during routine clinical care in NHS vascular clinics and multidisciplinary team meetings at participating sites. Patients scheduled for endovascular revascularisation who meet the inclusion criteria will be approached by local clinical teams. Eligible patients will be provided with verbal and written information about the trial and given sufficient time to consider participation.

Written informed consent will be obtained by trained research staff prior to any trial-specific procedures. Recruitment will be supported by site investigators, research nurses and the wider clinical team. Patient and public involvement and engagement (PPI and E) representatives have reviewed all participant-facing materials to ensure clarity and acceptability. Recruitment will be monitored by the coordinating team, with regular feedback provided to sites to maintain recruitment targets across all participating NHS trusts.

Participating NHS organisations/trusts (recruiting sites):

University Hospitals of Leicester NHS Trust.

Manchester University NHS Foundation Trust St George’s NHS Trust.

Hull University Teaching Hospitals NHS Trust.

University Hospitals of North Midlands.

St George’s University Hospitals NHS Foundation Trust.

Royal Free London NHS Foundation Trust.

#### Assignment of interventions and randomisation

1:1 electronic randomisation. Minimisation based on CKD stage, age, diabetes and tissue loss.

### Data collection, management and analysis

#### Data collection methods

Clinical and patient-reported data will be collected by trained research staff at each participating NHS site, supported by the coordinating Clinical Trials Unit (CTU). Data will be collected at baseline, during the index procedure and at follow-up visits at Days 2, 30 and 90, which coincide with routine NHS care.

Baseline data will include demographics, medical history, comorbidities, height, weight and baseline SCr. Procedural data will include details of the intervention, type and volume of contrast used, pain scores using a VAS and any immediate complications.

Follow-up data will include repeat SCr measurements, assessment of adverse events (AEs), MAKE, reinterventions, hospital stay duration and patient-reported outcome measures (EQ-5D-5L and SF-36) at Day 90. Healthcare resource use will be captured via case report forms (CRFs) and participant questionnaires to inform the health economic evaluation.

Optional qualitative data will be collected via semistructured interviews with a purposive sample of participants and practitioners to explore acceptability, feasibility and implementation factors. Interviews will be conducted by trained qualitative researchers following a topic guide.

All data will be entered into a secure, validated electronic data capture system managed by the CTU. Source data verification and monitoring will be conducted according to the trial monitoring plan and Sponsor standard operating procedures (SOPs).

#### Data management and monitoring

- Data will be collected via appropriate approved electronic CRFs and software (secure and encrypted) and managed by Leicester Clinical Trials Unit (LCTU).
- An independent Data Monitoring Committee (DMC) will oversee the trial.
- Serious AEs will be reported per NHS-established Good Clinical Practice guidelines.
- Monitoring as per Sponsor SOPs.

#### Trial management

The KID trial will be managed by the LCTU, a UK Clinical Research Collaboration-registered CTU (registration number 43), colocated with the chief investigator (CI) at the University of Leicester (UoL). The LCTU will provide trial management, statistical data and quality oversight support throughout the trial duration. The study is sponsored by the UoL.

Trial oversight will be provided through three key governance structures: the Trial Management Group (TMG), the Trial Steering Committee (TSC) and the DMC. The TMG will meet weekly or as needed and will be responsible for the day-to-day operational management of the trial. It will include the CI, coapplicants, the trial manager, the lead statistician and two lay coapplicants. The lay coapplicants will also liaise with an independent lay advisory group to ensure patient-centred conduct and delivery of the trial. The TSC will meet at least every 6 months. It will be chaired by an independent member and include external clinical, statistical and lay representatives. The TSC will provide high-level oversight, ensure adherence to the protocol and advise on trial progress and conduct.

The DMC will meet separately, prior to each TSC meeting. Comprising independent clinical and statistical experts, including an independent Chair, the DMC will review accumulating trial data with respect to participant safety and data integrity. It will make recommendations to the TSC regarding the continuation, modification or termination of the trial based on these reviews.

#### Data analysis

The LCTU Trial Statistician will be responsible for planning, monitoring and carrying out analyses, supervised by a senior statistician. Statistical analysis will be undertaken according to a prespecified statistical analysis plan (SAP) written prior to database lock and reported following Consolidated Standards of Reporting Trials statements. The baseline characteristics of the participants will be summarised by group. Continuous variables will be summarised using mean and SD, or median and IQR if data do not have a symmetric distribution. Categorical variables will be summarised with counts and percentages. The primary analysis of the primary outcome will be carried out in the ITT population using a mixed effects model with participant as a random effect to account for repeated measures over time. Each participant will contribute up to three repeated outcome measures to the model in addition to the baseline SCr. The model will be adjusted for the stratification and minimisation variables (age, sex, baseline CKD stage and presence of tissue loss). Secondary outcomes will be analysed on a complete case population and reported similarly to the primary outcome using appropriate methodology to compare randomised groups; binary outcomes using logistic regression, continuous outcomes using linear regression or, where not normally distributed, using appropriate non-parametric methods.

### Implementation and process evaluation

A mixed-methods explanatory design will explore the acceptability, feasibility and factors influencing implementation. The evaluation is informed by Proctor *et al*’s Implementation Outcome Framework, the TFA.

#### Patient participants

All trial participants will complete the 8-item TFA questionnaire at each assessment time point. Up to 12 participants will be purposively sampled for qualitative interviews 3–4 weeks postprocedure, conducted face-to-face or remotely by an experienced researcher using a semistructured, iterative guide codeveloped with PPI&E representatives.

#### Practitioner participants

All practitioners performing the procedures will complete online surveys (TFA, Intervention Appropriateness, Feasibility of Intervention Measure, Innovation-Specific Implementation Intentions) within 72 hours postprocedure. Up to 20 practitioners will be purposively sampled for qualitative interviews exploring training, delivery and implementation barriers/facilitators. Interviews will be transcribed and analysed thematically using TFA domains, with participant checking and PPI&E/advisory input to contextualise findings.

### Health economic evaluation

A microcosting analysis will capture intraprocedural costs, including devices, consumables and staff time. Additional NHS and community health service use will be collected over 90 days, alongside EQ-5D-5L for Quality Adjusted Life Years (QALY) estimation. Secondary analyses will include productivity losses. Where appropriate, long-term outcomes will be modelled using a state-transition approach. Sensitivity analyses will assess uncertainty. All analyses will follow National Institute for Health and Care Excellence (NICE) and Consolidated Health Economic Evaluation Reporting Standards guidelines.

### Measurement of costs and outcomes

Health economic analysis includes direct and indirect costs.

### Statistical methods

#### Planned analyses for primary and secondary outcomes

The primary analysis will be performed on an ITT basis, including all randomised participants as allocated. The primary outcome—log SCr at 2, 30 and 90 days postprocedure—will be analysed using a mixed-effects model with participant as a random effect to account for repeated measures over time. The model will adjust for baseline log SCr, stratification and minimisation factors (age, sex, baseline CKD stage and presence of tissue loss).

Secondary outcomes will be analysed using appropriate statistical methods: binary outcomes (eg, AKI incidence, MAKE) will be compared between groups using logistic regression; continuous outcomes (eg, contrast volume, QoL scores) will be analysed using linear regression, or non-parametric tests if data are not normally distributed. Time-to-event data, if applicable, will be analysed using survival analysis methods such as Kaplan-Meier estimates and Cox proportional hazards regression. All analyses will be conducted using validated statistical software according to a prespecified SAP.

#### Handling of missing data

Missing outcome data will be assessed for extent and patterns. For the primary outcome, multiple imputation using a multivariate normal regression model will be applied if data are missing at random, with results pooled across imputed datasets. Sensitivity analyses will be conducted to evaluate the impact of different assumptions about missing data. Secondary outcomes will be analysed on a complete case basis, unless otherwise specified.

### Ethics and dissemination

The trial has received full ethical approval from an NHS Research Ethics Committee (reference: 24/WA/0332). All amendments, including Non Substantive Amendment (NSA)01 (clarifying protocol deviations, randomisation/recruitment timelines, introduction of the compiled questionnaire booklet and secure interview transcription method) and NSA02 (clarifying inclusion criteria for CKD participants) have been reviewed and approved to ensure continued compliance with regulatory requirements.

Written informed consent will be obtained from all participants before any trial-specific procedures take place. Consent will include agreement for participation in the main trial and any optional qualitative substudy components. Participant information sheets and informed consent forms have been updated to reflect changes described in the approved amendments, including the use of secure, encrypted transcription devices for interviews and the introduction of the compiled questionnaire booklet. All participant information will be kept strictly confidential and handled in line with the General Data Protection Regulation and the UK Data Protection Act. Personal identifiers will be stored securely at sites and not transferred to the trial database. Audio recordings of interviews will be encrypted and securely transcribed using a standalone device to prevent unauthorised access. Practitioner identifiers have been updated in line with data protection best practice.

Trial results will be disseminated through publication in peer-reviewed journals and presentations at national and international conferences (eg, American Vascular Society conference, European Society for Vascular Surgery conference). Lay summaries and updates will be shared through lay collaborators, community networks and social media channels. A hybrid event for patients and the public will be organised to present key findings, supported by accessible outputs such as infographics, video summaries and a dedicated trial webpage hosted by Vascular Research UK.

### Access to data

Access to the final trial dataset will be limited to authorised trial team members only; however, anonymous data will be shared based on Health Research Authority guidance with scientific collaborators who can/will contact the study investigators. Any data sharing will be in anonymised form and compliant with participant consent and applicable data protection legislation.

### PPI&E before and during trial delivery

PPI&E representatives are involved with design, delivery and dissemination strategies. Three lay focus groups have been convened to develop this trial. 10 patients who had endovascular PAD treatment previously, two full-time carers and two partners took part. We explored outcomes of interest, follow-up procedures and clinically important differences. We gave participants multiple options to choose from regarding all of the above. We also asked them to volunteer their own views/opinions both in open discussions and individual face-to-face interviews. Two formal PPI&E coapplicants, a female and a male patient (different ethnic backgrounds) who have had PAD procedures, support this trial. They will join all trial meetings, review trial materials and support dissemination. The two PPI&E coapplicants will lead the final dissemination event, which we will host on completion of the trial, to share findings with patient networks and NHS staff nationally.

## Discussion

The KID trial addresses a critical and currently unmet need in the management of patients with PAD and CKD undergoing endovascular intervention (1). AKI is a common and serious complication in this population, largely attributed to the use of iodinated contrast medium. This multicentre, RCT will evaluate whether the use of CO_₂_ delivered via an automated injector (Angiodroid) can reduce the incidence and severity of renal impairment in the short and medium term, compared with standard iodinated contrast.

While previous observational studies have indicated a potential benefit of CO_₂_ angiography in preserving renal function, this will be the first trial to evaluate its use systematically in a PAD/CKD cohort using robust clinical, implementation and health economic endpoints. Incorporating a mixed-methods process evaluation and qualitative analysis ensures that acceptability, feasibility and implementation barriers are explored in both patients and practitioners.

Several practical and operational challenges are anticipated. These include variation in operator experience with CO_₂_ delivery, ensuring high-quality imaging and managing procedural discomfort, which some patients may experience. Further, CO_₂_ injectors are not yet widely used across all NHS centres, requiring site-specific support and training. Patient follow-up at three separate time points (2, 30 and 90 days) also presents logistical demands, particularly in a population that may have mobility or access issues.

Nonetheless, the trial is strengthened by its pragmatic design, national recruitment strategy and the use of existing NIHR infrastructure. A data management plan and engagement with experienced vascular centres will help ensure consistency in intervention delivery and data collection. The trial has been codeveloped with extensive PPI&E, ensuring relevance of outcomes and acceptability of follow-up protocols.

If successful, the KID trial could support a shift in clinical practice by providing definitive evidence on the utility of automated CO_₂_ angiography in reducing AKI, potentially influencing NICE guidelines and NHS resource allocation. A reduction in AKI incidence could not only improve clinical outcomes but also yield substantial cost savings for the healthcare system. The findings will therefore have significant implications for both clinical and policy decision-making in vascular care.
